# Modelling the association between fibrinogen concentration on admission and mortality in patients with massive transfusion after severe trauma: an analysis of a large regional database

**DOI:** 10.1186/s13049-018-0523-0

**Published:** 2018-07-09

**Authors:** Pierre Bouzat, François-Xavier Ageron, Jonathan Charbit, Xavier Bobbia, Pauline Deras, Jennifer Bas Dit Nugues, Etienne Escudier, Guillaume Marcotte, Marc Leone, Jean-Stéphane David

**Affiliations:** 1 Grenoble Alps Trauma center, Department of anesthesiology and intensive care medicine, Grenoble University Hospital, F-38000 Grenoble, France; 2 Grenoble Alps University, F-38000 Grenoble, France; 3 RENAU Northern French Alps Emergency Network, Public Health department, Annecy Hospital, F-74000 Annecy, France; 4 Department of emergency medicine and intensive care, Annecy Hospital, F-74000 Annecy, France; 5 Department of anesthesiology and intensive care, Montpellier University Hospital, F-34000 Montpellier, France; 6 Department of emergency medicine, Nimes University Hospital, F-30000 Nimes, France; 7 Department of anesthesiology and intensive care, Lyon-Edouard Herriot University Hospital, F-69000 Lyon, France; 8 Aix Marseille University, Nord Hospital, Department of anesthesiology and intensive medicine, APHM, F-13000 Marseille, France; 9 Hospices Civils de Lyon, Lyon-Sud University Hospital, Department of anesthesiology and intensive care, F-69495 Pierre-Bénite, France; 10 Claude Bernard Lyon 1 University, F-69008 Lyon, France; 11 Pôle d’Anesthésie-Réanimation, Hôpital Albert Michallon, 217, F-38043 Grenoble, BP France

**Keywords:** Fibrinogen, Mortality, Massive transfusion, Severe trauma

## Abstract

**Background:**

The relationship between fibrinogen concentration and traumatic death has been poorly explored after severe trauma. Existing studies analysed this relationship in unselected trauma population, often considering fibrinogen concentration as a categorical variable. The aim of our study was to model the relationship between fibrinogen concentration and in-hospital mortality in severe trauma patients requiring massive transfusion using fibrinogen on admission as a continuous variable.

**Methods:**

We designed a retrospective observational study based on prospectively collected data from 2009 to 2015 in seven French level-I trauma centres. All consecutive patients requiring a transfusion of at least 10 packed red blood cells (RBC) within 24 h were included. To assess the relationship between in-hospital death and fibrinogen concentration on admission, we performed generalized linear and additive models with death as a dependent variable. We also assessed the relationship between fibrinogen concentration below 1.5 g.L^− 1^ and potential predictors.

**Results:**

Within the study period, 366 patients were included. A non-linear relationship was found between fibrinogen concentration and death. Graphical modelling of this relationship depicted a negative association between fibrinogen levels and death below a fibrinogen concentration of 1.5 g.L^− 1^. Predictors of low fibrinogen concentration (< 1.5 g.L^− 1^) were systolic blood pressure, Glasgow coma scale and haemoglobin concentration on admission.

**Conclusions:**

A complex and robust approach for modelling the relationship between fibrinogen and mortality revealed a critical fibrinogen threshold of 1.5 g.L^− 1^ for severe trauma patients requiring massive transfusion. This trigger may guide the administration of procoagulant therapies in this context.

## Introduction

Fibrinogen is a glycoprotein involved in the final process of the coagulation cascade. This protein is the precursor of fibrin that binds with platelets for clot formation [1]. Early decrease in fibrinogen concentration was found in patients with on-going bleeding after severe trauma suggesting a critical role for this coagulation factor in such setting [2, 3]. Other authors demonstrated a potential relationship between fibrinogen concentration and mortality in large unselected trauma population [4–6]. Based on this evidence, European guidelines recommend maintaining fibrinogen level between 1.5 and 2 g.L^− 1^ [7]. However, there is still uncertainty regarding the relationship between fibrinogen concentration and death during the management of severe trauma patients with active bleeding since different critical concentrations for fibrinogen have been reported between 1 and 2 g.L^− 1^ using different statistical approaches [4, 5, 8].

Apart from beneficial effects, aggressive correction of fibrinogen level can also induce a pro-thrombotic state with potential thrombo-embolic events. Since post-traumatic coagulopathy is characterized by a rapid shift from an anti-thrombotic to a pro-thrombotic state [9], over-correction of fibrinogen concentration may promote delayed severe side-effects in intensive care unit. Accordingly, Wafaisade et al. reported a trend towards an increase in thrombo-embolic events in patients receiving fibrinogen therapy as compared to patients without fibrinogen administration [10]. Taken together, these data indicate a need for an accurate selection of patients requiring fibrinogen correction on admission based on a robust threshold [11]. The relevancy of such threshold depends on whether patients had massive haemorrhage or not since fibrinogen concentration in patients without significant bleeding is not relevant. As a result, the description of the relationship between fibrinogen concentration on admission and death in a selected trauma population requiring massive transfusion would help determining the threshold to trigger the administration of procoagulant therapies on admission. We, thus, designed a retrospective study based on existing regional registries to model the relationship between fibrinogen concentration and death in patients who received a massive transfusion within the first 24 h. Our hypothesis is that fibrinogen concentration has a non-linear relationship with in-hospital death in these patients, allowing the determination of the fibrinogen threshold for increased risk of death.

## Patients and methods

### Study design and selection criteria

We designed a retrospective observational study based on prospectively collected data from 2009 to 2015 by trauma registries in seven French Level I trauma centres (Grenoble Alps trauma centre, Annecy, Montpellier, Nimes, Marseille (Hôpital Nord), Lyon-Sud and Lyon-Edouard Herriot hospital). We included consecutive trauma patients who received a massive transfusion defined by a transfusion of at least 10 packed red blood cells (RBC) within the first 24 h following the admission. Exclusion criteria were patients under 15-year-old. The design of the study received ethical approval by the French Society of Anaesthesiology and Intensive care (IRB number: CERAR 00010254–2017-052). According to the French law No. 2012–300 (Decree No. 2016–1537), non-opposition of surviving patients was obtained by an information letter, and the declaration was made to the French Data Protection Authority.

### Data collection

For each patient, we collected demographic data: age, gender, type of injury, Injury Severity Score (ISS), Mechanism, Glasgow, Age and systolic arterial blood Pressure (MGAP) score, transfusion requirement with RBC, Fresh Frozen Plasma (FFP) and Platelet (PLT), and the type of haemostatic treatment. Vital parameters including systolic arterial blood pressure (SBP), heart rate, pulse oximetry and Glasgow coma scale (GCS) were also reported on admission. Morbidity and mortality assessment consisted of death at 28-day, length of stay in intensive care unit (ICU) and length of stay in hospital. We also collected biologic data on admission: fibrinogen concentration, prothrombin time, haemoglobin, platelet count and lactate concentration.

### Outcome and exposure

The primary endpoint was in-hospital death in order to model the relationship between fibrinogen concentration and death.

Secondary endpoint was low fibrinogen concentration at hospital admission (< 1.5 g.L^− 1^) in order to explore the relationship between low fibrinogen concentration and potential predictors on admission (systolic blood pressure, heart rate, Glasgow coma scale, age, gender, haemoglobin concentration).

### Statistical analysis

Baseline patient characteristics were reported as medians and interquartile range (IQR 25th–75th) or means and standard deviation when appropriate for continuous variables. Categorical variables were reported as frequency and percentages.

To assess the relationship between death and fibrinogen concentration on hospital admission, we performed a Generalized Linear Model (GLM) with death as a dependent variable. We graphically assessed the departure from linearity and include quadratic and cubic terms in the equation to perform a polynomial regression model. We removed, one at a time, each polynomial term for which there was no evidence for an association based on the *P* values (< 0.05) from the Wald test. Each time, we performed a likelihood ratio test between models to ensure that removal of the variable did not affect the model based on the P value (> 0.05). As we expected a nonlinear relationship between death and fibrinogen concentration, we performed in addition a Generalized Additive Model (GAM). GAM is a flexible method with a smooth function based on cubic spline [12]. It allows characterizing nonlinear relationship of exposure on outcome. We graphically assessed the relationship between death and fibrinogen concentration on admission by plotting the predicted risk of death on fibrinogen concentration. The threshold value for fibrinogen concentration corresponded to the lowest value of predicted risk of death. We assessed the performance of the GLM in term of discrimination using the area under the curve (AUC), the sensibility and specificity of the threshold value and in term of calibration using the calibration-in-the-large and the calibration slope.

To assess the relationship between low fibrinogen concentration and potential predictors, we used the threshold value estimated previously. We selected all variables available on hospital admission: age, gender, penetrating or blunt injury, SBP, heart rate and GCS. Haemoglobin concentration was also included in the variable selection as routinely available at the bedside. We performed a GLM with low fibrinogen concentration as dependent variable. We used the same method as the previous model for modelling continuous variables using linear and polynomial terms. In addition, we performed a LASSO method (Least absolute Shrinkage and Selection Operator) for variable selection of the model [13].

We tested all plausible interaction in the different models. We assessed overall performance of the different models with the Brier score and the discrimination with concordance (or C) statistic, the sensitivity and specificity of the threshold value. The calibration was assessed by the calibration-in-the-large and the calibration slope. We also included in the different models a random effect on trauma centre to control for clustering by using mixed-effects GLM and mixed-effects GAM.

Regarding missing data, outcome was missing for one patient. Fibrinogen concentration on admission was missing for 42 patients (11%). We assumed that missing data were at random. We performed multiple imputation by chained equation. We generated 20 datasets of 366 observations to fill in missing values for fibrinogen concentration on admission, systolic blood pressure, heart rate, Glasgow coma scale and haemoglobin on admission.

Two-sided *p*-values lower than 0.05 were considered statistically significant. All analyses were performed using Stata version 14.0 (Stata Corporation, College Station, TX, USA) and R software version 3.4.3 using mgcv package (R Foundation).

## Results

**Fig. 1:**
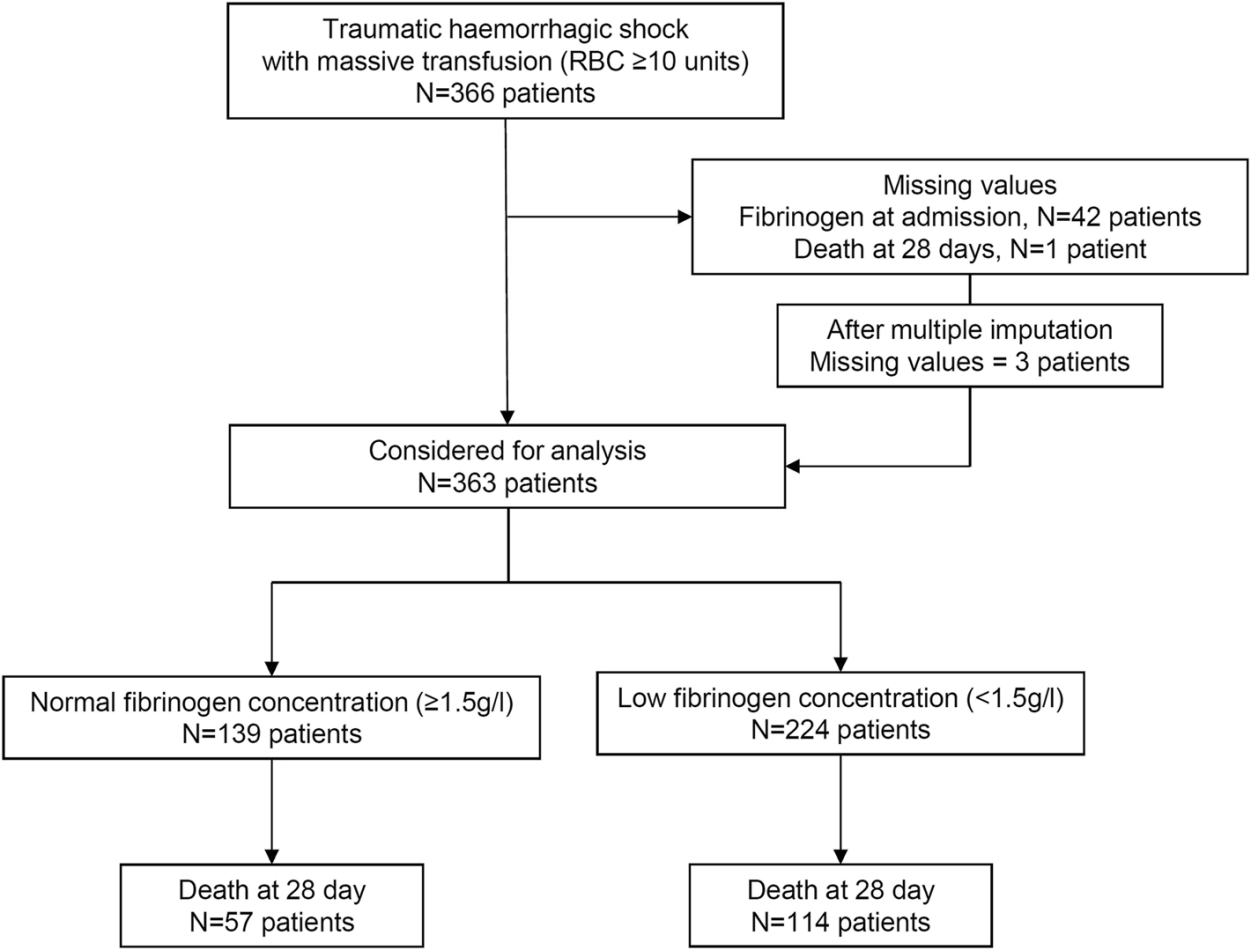
Flow chart of the study

Within the study period, 366 consecutive patients received at least 10 RBCs during the first 24 h. We estimated that approximately 11,550 severe trauma patients (ISS > 15) were admitted in the seven trauma centres within the same study period. Flow chart of included patients is presented in Fig. 1. Main demographic characteristics of the trauma population are presented in Table 1. Fibrinogen concentration on admission was missing for 42 patients (11%). Other predictors were missing from 3% up to 9% (Table 1). We imputed 118 missing values out of 1830 values (6%) to analyse 363 patients. Blood products and haemostatic procedures are reported in Table 2. The median fibrinogen concentration on admission was 1.1 g.L^− 1^ [0.6–1.7]. At Day 28, 174 patients (48%) did not survive. Median duration of ICU stay was 6 [1–20] days and median duration of hospital stay was 16 [1–37] days.

**Table 1:** Characteristics of the 366 patients with a massive transfusion following severe trauma

	Value	Missing (%)
Age years, mean (sd)	42 (18)	0
Male, n (%)	241 (75)	0
Injury Severity Score, mean (sd)	41 (17)	0.3
MGAP score, mean (sd)	20 (6)	–
Penetrating injury, n(%)	30 (9)	0
Mechanism of injury, n (%)		0
Road traffic accident	185 (57)	–
Fall	57 (18)	–
Others	42 (13)	–
Vital parameters at admission		
Heart rate (bpm), mean (sd)	102 (34)	3.4
Systolic blood pressure (mmHg), mean (sd)	88 (35)	2.7
Glasgow coma scale, n(%)		5.8
3–8	140 (46)	–
9–12	24 (8)	–
13–15	140 (46)	–
Biologic parameters at admission, Median [IQR]		
Fibrinogen concentration (g/l)	1.1 [0.6–1.7]	0
Prothrombin time (%)	40 [28–58]	1.2
Platelet count (g/l)	137 [91–196]	4.6
Haemoglobin (g/l)	91 [74–111]	3.4
Lactate (mmol/l)	5.7 [3.2–9.5]	24.1

*Sd* Standard deviation, *ISS* Injury Severity Score, *MGAP* Mechanism, Glasgow, Age, systolic blood Pressure, *Bpm* Beats per minute, *IQR* Interquartile range

**Table 2:** Blood products and haemostatic procedures

Blood products within the first 24 h	Median [IQR]
Red Blood Cells (Unit)	14 [11–19]
Fresh Frozen Plasma (Unit)	12 [8–16]
Platelet (Unit)	2 [1–3]
Fibrinogen concentrate (gr)	4.5 [3–7.5]
Emergency procedures	N (%)
Surgery for bleeding	206 (64)
Embolization	83 (26)

*IQR* Interquartile Range

**Fig. 2:**
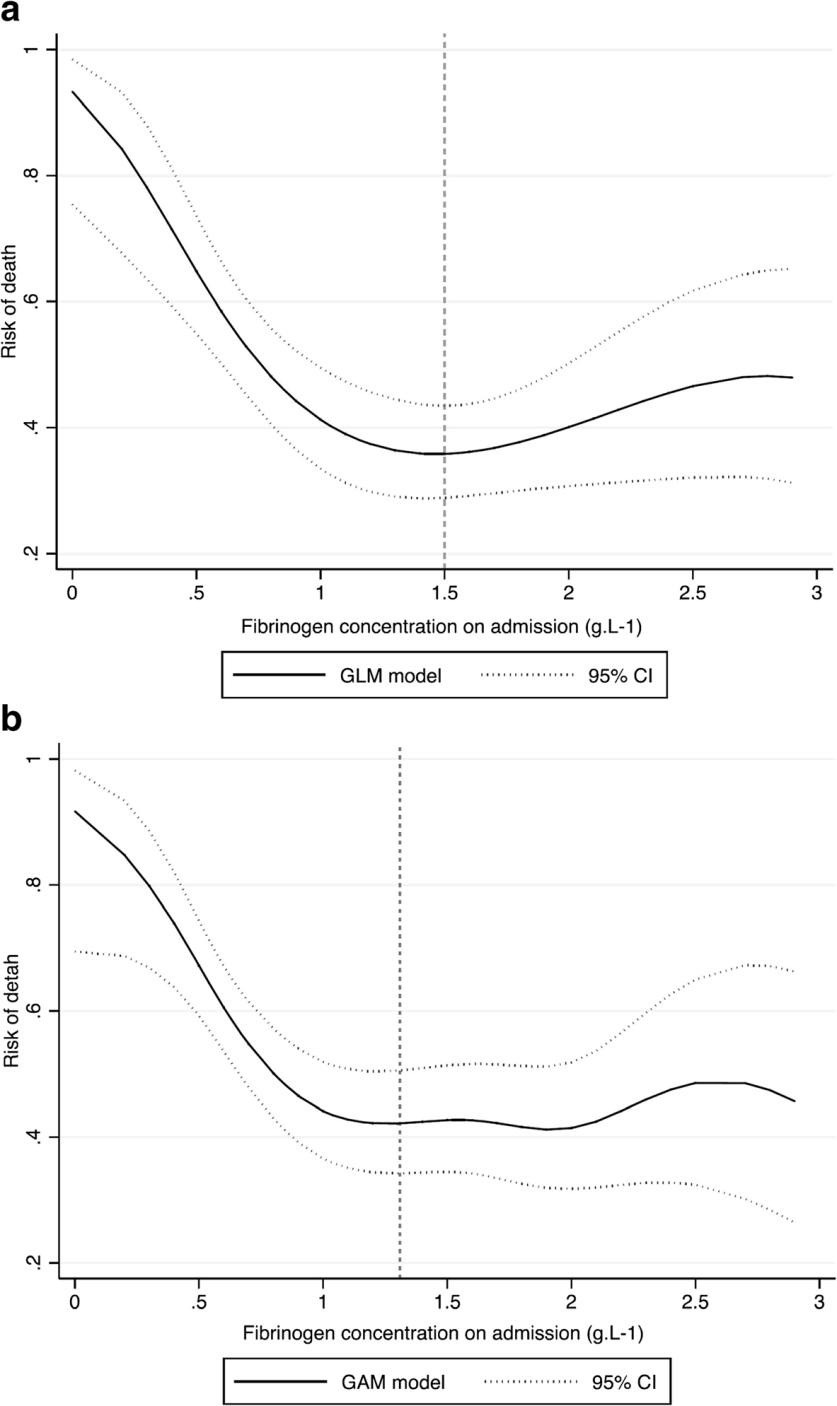
Modelling risk of death according to fibrinogen concentration: **a**. Generalized linear model (GLM) and **b**. Generalized additive model (GAM). Footnotes Fig. 2 (**a**) Mixed-Generalized Linear Model (GLM) with random effect on trauma center. Polynomial regression including quadratic and cubic terms. *P* value for linear term = 0.002; *N* = 363; C-stat = 0.62, Brier = 0.23; Predicted risk of death lowest value for fibrinogen concentration of 1.50 (**b**) Mixed-Generalized Additive Model (GAM) with random effect on trauma center and 6 equivalent degree of freedom (P value = 0.008). N = 363; Predicted risk of death lowest value for fibrinogen concentration of 1.31

The relationship between death and fibrinogen concentration showed a statistically significant non-linear association in both model: polynomial regression GLM (Fig. 2a) and GAM (Fig. 2b). Both model showed a strong negative association. The lowest value of the predicted risk of death was for a fibrinogen concentration of 1.5 g.L^− 1^ and 1.3 g. L^− 1^, respectively for the GLM and GAM. C-statistic was 0.63; 95% CI (0.57–0.69). The sensitivity for a fibrinogen concentration of 1.5 g.L^− 1^ was 98% and a specificity of 3%. The ratio of the predicted risk of death and observed death was 1.06; 95%CI (0.95–1.17) showing an adequate calibration. Calibration slope was 1.2; 95%CI (0.74–1.74) showing not overfitting.

**Fig. 3:**
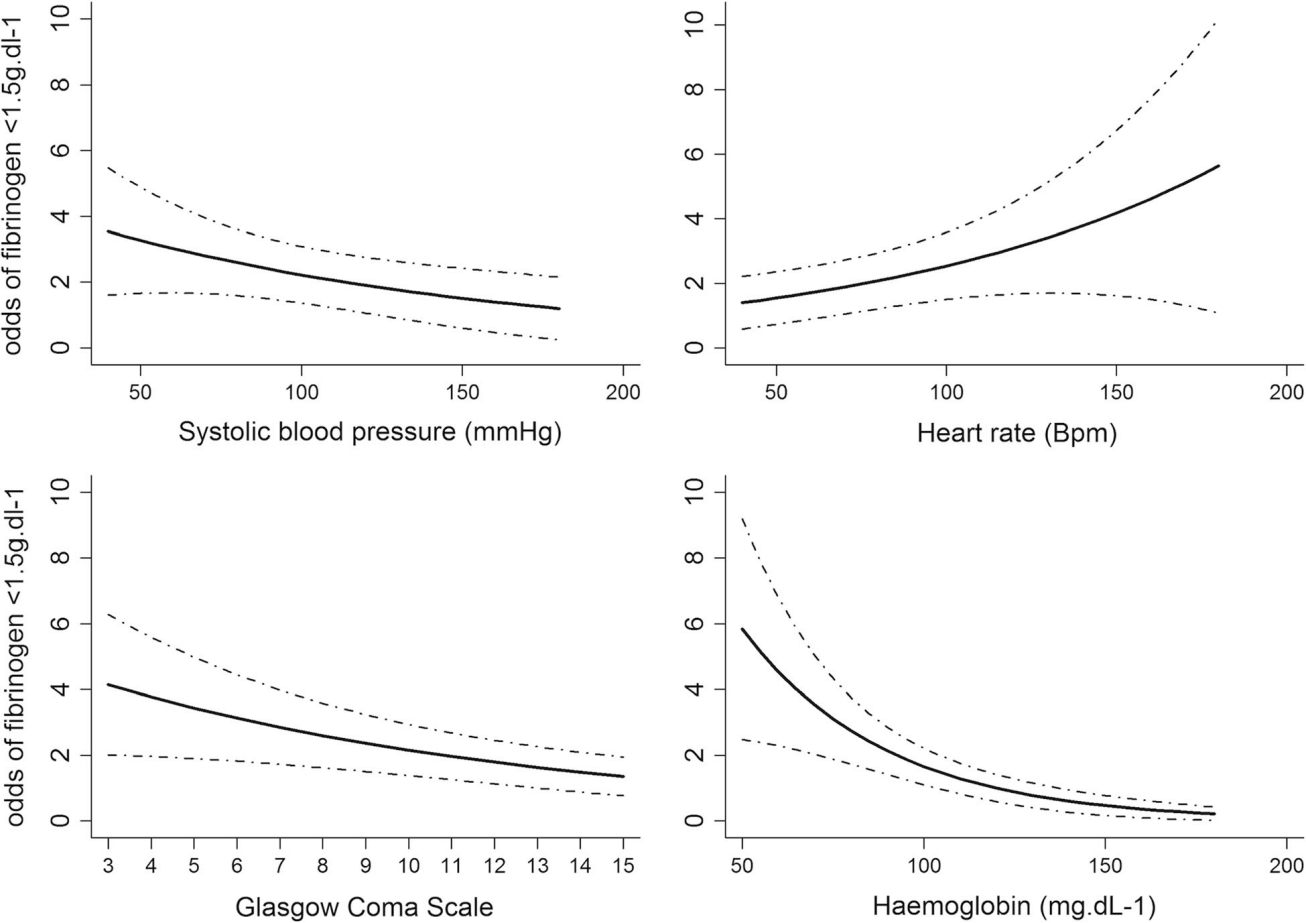
Predictors of low fibrinogen concentration in the generalized linear model (GLM). Each graph represents the relationship between a predictor (systolic blood pressure, heart rate, Glasgow coma scale and haemoglobin) and the odds of fibrinogen concentration < 1.5 g.L^− 1^. Footnotes Fig. 3 Mixed-Generalized Linear Model with random effect on trauma center; *N* = 362, C-stat = 0.75 95%CI (0.69–0.81), Brier score = 0.19

Two hundred twenty four patients (62%) had a fibrinogen concentration lower than 1.5 g.L^− 1^ on admission. The Table 3 reports the association between low fibrinogen concentration and predictors at baseline (hospital admission). Three predictors were associated to low fibrinogen concentration (Fig. 3). Systolic blood pressure, Haemoglobin and GCS showed a linear negative association with fibrinogen < 1.5 g.L^− 1^. Heart rate, age, gender and mechanism of injury were not associated to low fibrinogen concentration. Penalized regression model with LASSO did not show any difference in the coefficient (less than 5% of relative difference) and in the variable selection.

**Table 3:** Predictors at hospital admission, multivariate analysis using Generalized Linear Model (GLM)

	OR (95%CI)	P value
Heart rate	1.01 (1.00–1.02)	0.093
Systolic blood pressure	0.99 (0.98–1.00)	0.064
Glasgow coma scale	0.92 (0.88–0.97)	0.002
Haemoglobin level	0.98 (0.97–0.99)	< 0.001
Age	0.99 (0.97–1.00)	0.118
Sex male	0.81 (0.35–1.84)	0.596
Penetrating injury	0.80 (0.30–1.79)	0.494

Mixed-Generalized Linear Model; N = 362, C-stat = 0.74 95%CI (0.69–0.80), Brier score = 0.19

## Discussion

In a cohort of consecutive trauma patients with active bleeding requiring massive transfusion, we observed a non-linear association between fibrinogen concentration on admission and death. A strong negative association between these parameters was observed below the fibrinogen threshold of 1.5 g.L^− 1^. Predictors for fibrinogen concentration lower than 1.5 g.L^− 1^ on admission were classic vital parameters on admission: systolic blood pressure, Glasgow coma scale and haemoglobin. Our work highlights the role of fibrinogen in critically-bleeding patients after severe trauma and further confirms the increase in the risk of death below a fibrinogen concentration of 1.5 g.L^− 1^ on admission.

Low fibrinogen concentrations have been frequently described on admission and during the initial management of severe trauma patients [11]. More importantly, subsequent decrease in fibrinogen concentration has been associated with injury severity and post-traumatic coagulopathy [14]. In our study, we also evaluated the association between fibrinogen on admission and death. Previously, Hagemo et al. showed a nonlinear relationship between fibrinogen concentration and death using a multicentre cohort of 1133 unselected trauma patients [4]. The incidence of low fibrinogen concentration in this study was low since only 93 (8.2%) patients had a fibrinogen value lower than 1.5 g.L^− 1^. This study also included non-haemorrhagic patients and identified a higher critical threshold of 2.3 g.L^− 1^. Inaba et al. also reported a statistical association between fibrinogen concentration and death in a single-centre cohort of 260 trauma patients with massive transfusion [8]. In this study, the authors stratified patients in three categories following their fibrinogen levels on admission. As a result, fibrinogen was not considered as a continuous variable, which induced a considerable bias to analyse the relationship between fibrinogen and mortality. A critical threshold was identified at 1 g.L^− 1^ by this study. More recently, using an Australian state-wide registry with 4773 patients, initial fibrinogen levels sorted in three categories were also independently associated with in-hospital death [5]. Using a more robust and complex analysis for continuous variables, we found a threshold of 1.5 g.L^− 1^ in patients requiring massive transfusion after severe trauma. Strengths of the present study are i) the limitation of misclassification and false estimate by a non-categorical approach for fibrinogen levels and ii) a homogenous population of severe bleeding patients. We also used a prospective inception cohort starting at hospital admission. Finally, this study was based on data collected from seven trauma centres that allows more generalizable results than a single-centre study.

Predictors for low fibrinogen concentration were classic vital parameters on admission such as heart rate, systolic arterial blood pressure, Glasgow coma scale and haemoglobin. Recently a trauma score, named FibAT, was created to predict a fibrinogen level lower than 1.5 g.L^− 1^ [15]. Predictors were identified using a derivation cohort of 2124 patients and consisted of age, heart rate, SBP, blood lactate and haemoglobin. We further confirmed these predictors except for age. Although baseline fibrinogen levels depend on age [16], the pathophysiology of fibrinolysis and the loss of coagulation factors are conversely independent from age [17]. We thus believe that the threshold for increased the risk of death is not different between a young and an old trauma population. Potential mechanisms responsible for low fibrinogen concentration on admission might be the activation of fibrinolysis (due to the severity of injury and/or on-going tissue hypoperfusion) and the loss of coagulation factors by active bleeding [1, 18, 19].

One important issue when managing trauma patients with active bleeding is the establishment of threshold to trigger the administration of procoagulant therapies [20, 21]. With the present study, we suggest that a fibrinogen concentration of 1.5 g.L^− 1^ is critical in patients requiring massive transfusion after severe trauma. This finding could be seen as a target to guide the administration of fibrinogen concentrate during resuscitation. More importantly, this threshold may help the design of future studies using fibrinogen concentrates. Indeed, there is still uncertainty regarding fibrinogen targets when designing future trials assessing the correction of fibrinogen deficit in post-traumatic coagulopathy [20]. Our study indicates that a fibrinogen level of 1.5 g.L^− 1^ is probably the target to be reached in the intervention group.

We acknowledge several limits of our study. We conducted a retrospective analysis of prospectively collected data and we did not perform a prospective trial per se. As a consequence, there is no randomization and we cannot account for potential unknown confounders. Finally, missing values are inherent to retrospective studies based on on-going registries. However, missing values did not interest the main outcome (death) since only one value was missing. Moreover, we performed multiple imputations to manage missing values for predictors. We assumed that missing values were at random. If not, this could lead to bias toward the null. In addition of a probably lack of power, this could explain the weak association between systolic blood pressure, heart rate and low fibrinogen concentration.

## Conclusion

Low fibrinogen concentration on admission was associated with an increase in the risk of death in severe trauma patients requiring massive transfusion. The threshold of 1.5 g.L^− 1^ was found for a negative relationship between fibrinogen levels and in-hospital death. Classic vital parameters were independent predictors of low fibrinogen concentration on admission. Our study also provides information regarding the design of future potential trials about fibrinogen supplementation after severe trauma, suggesting a threshold of 1.5 g.L^− 1^ for fibrinogen concentrate therapy and may also guide the administration of procoagulant therapies.

## References

[1] Chang R, Cardenas JC, Wade CE, Holcomb JB (2016). Advances in the understanding of trauma-induced coagulopathy. Blood.

[2] Floccard B, Rugeri L, Faure A, Saint Denis M, Boyle EM, Peguet O, Levrat A, Guillaume C, Marcotte G, Vulliez A (2012). Early coagulopathy in trauma patients: an on-scene and hospital admission study. Injury.

[3] Rourke C, Curry N, Khan S, Taylor R, Raza I, Davenport R, Stanworth S, Brohi K (2012). Fibrinogen levels during trauma hemorrhage, response to replacement therapy**,** and association with patient outcomes. Journal of thrombosis and haemostasis : JTH.

[4] Hagemo JS, Stanworth S, Juffermans NP, Brohi K, Cohen M, Johansson PI, Roislien J, Eken T, Naess PA, Gaarder C (2014). Prevalence, predictors and outcome of hypofibrinogenaemia in trauma: a multicentre observational study. Crit Care..

[5] McQuilten ZK, Wood EM, Bailey M, Cameron PA, Cooper DJ (2017). Fibrinogen is an independent predictor of mortality in major trauma patients: a five-year statewide cohort study. Injury.

[6] Hess JR, Lindell AL, Stansbury LG, Dutton RP, Scalea TM (2009). The prevalence of abnormal results of conventional coagulation tests on admission to a trauma center. Transfusion.

[7] Rossaint R, Bouillon B, Cerny V, Coats TJ, Duranteau J, Fernandez-Mondejar E, Filipescu D, Hunt BJ, Komadina R, Nardi G (2016). The European guideline on management of major bleeding and coagulopathy following trauma: fourth edition. Critical care (London, England).

[8] Inaba K, Karamanos E, Lustenberger T, Schochl H, Shulman I, Nelson J, Rhee P, Talving P, Lam L, Demetriades D (2013). Impact of fibrinogen levels on outcomes after acute injury in patients requiring a massive transfusion. J Am Coll Surg.

[9] Curry NS, Davenport RA, Hunt BJ, Stanworth SJ (2012). Transfusion strategies for traumatic coagulopathy. Blood Rev.

[10] Wafaisade A, Lefering R, Maegele M, Brockamp T, Mutschler M, Lendemans S, Banerjee M, Bouillon B, Probst C (2013). Trauma registry of DGU: administration of fibrinogen concentrate in exsanguinating trauma patients is associated with improved survival at 6 hours but not at discharge. The journal of trauma and acute care surgery.

[11] Maegele M, Zinser M, Schlimp C, Schochl H, Fries D (2015). Injectable hemostatic adjuncts in trauma: fibrinogen and the FIinTIC study. The journal of trauma and acute care surgery.

[12] Hastie T, Tibshirani R (1995). Generalized additive models for medical research. Stat Methods Med Res.

[13] Friedman J, Hastie T, Tibshirani R (2010). Regularization paths for generalized linear models via coordinate descent. J Stat Softw.

[14] Rugeri L, Levrat A, David JS, Delecroix E, Floccard B, Gros A, Allaouchiche B, Negrier C (2007). Diagnosis of early coagulation abnormalities in trauma patients by rotation thrombelastography. Journal of thrombosis and haemostasis : JTH.

[15] Gauss T, Campion S, Kerever S, Eurin M, Raux M, Harrois A, Paugam-Burtz C, Hamada S, Traumabase G. Fibrinogen on admission in trauma score: early prediction of low plasma fibrinogen concentrations in trauma patients. Eur J Anaesthesiol. 2017; 10.1097/EJA.0000000000000734 29120938

[16] Ohmori T, Kitamura T, Tanaka K, Saisaka Y, Ishihara J, Onishi H, Nojima T, Yamamoto K, Matusmoto T, Tokioka T (2015). Admission fibrinogen levels in severe trauma patients: a comparison of elderly and younger patients. Injury.

[17] Madurska MJ, Sachse KA, Jansen JO, Rasmussen TE, Morrison JJ (2018). Fibrinolysis in trauma: a review. European journal of trauma and emergency surgery : official publication of the European Trauma Society.

[18] Martini WZ (2009). Coagulopathy by hypothermia and acidosis: mechanisms of thrombin generation and fibrinogen availability. J Trauma.

[19] Gall LS, Brohi K, Davenport RA (2017). Diagnosis and treatment of Hyperfibrinolysis in trauma (a European perspective). Semin Thromb Hemost.

[20] Samama CM, Ickx B, Ozier Y, Steib A, Susen S, Godier A. The place of fibrinogen concentrates in the management of perioperative bleeding: a position paper from the francophone working group on perioperative Haemostasis (GIHP). Anaesthesia, critical care & pain medicine. 2018; 10.1016/j.accpm.2018.04.002 29660502

[21] Winearls J, Campbell D, Hurn C, Furyk J, Ryan G, Trout M, Walsham J, Holley A, Shuttleworth M, Dyer W (2017). Fibrinogen in traumatic haemorrhage: a narrative review. Injury.

